# Improved Metaheuristics-Based Clustering with Multihop Routing Protocol for Underwater Wireless Sensor Networks

**DOI:** 10.3390/s22041618

**Published:** 2022-02-18

**Authors:** Prakash Mohan, Neelakandan Subramani, Youseef Alotaibi, Saleh Alghamdi, Osamah Ibrahim Khalaf, Sakthi Ulaganathan

**Affiliations:** 1 Department of Computer Science and Engineering, Karpagam College of Engineering, Coimbatore 641032, India; 2 Department of Computer Science and Engineering, R.M.K Engineering College, Chennai 601206, India; snksnk17@gmail.com; 3 Department of Computer Science, College of Computer and Information Systems, Umm Al-Qura University, Makkah 21955, Saudi Arabia; yaotaibi@uqu.edu.sa; 4 Department of Information Technology, College of Computers and Information Technology, Taif University, Taif 21944, Saudi Arabia; s.algamedi@tu.edu.sa; 5 Al-Nahrain Nano Renewable Energy Research Center, Al-Nahrain University, Baghdad 10071, Iraq; usama.ibrahem@coie-nahrain.edu.iq; 6 Department of Computer Science and Engineering Saveetha School of Engineering, SIMATS, Chennai 602105, India; sakthiu.sse@saveetha.com

**Keywords:** underwater sensor networks, energy efficiency, metaheuristics, network lifetime, communication, routing

## Abstract

Underwater wireless sensor networks (UWSNs) comprise numerous underwater wireless sensor nodes dispersed in the marine environment, which find applicability in several areas like data collection, navigation, resource investigation, surveillance, and disaster prediction. Because of the usage of restricted battery capacity and the difficulty in replacing or charging the inbuilt batteries, energy efficiency becomes a challenging issue in the design of UWSN. Earlier studies reported that clustering and routing are considered effective ways of attaining energy efficacy in the UWSN. Clustering and routing processes can be treated as nondeterministic polynomial-time (NP) hard optimization problems, and they can be addressed by the use of metaheuristics. This study introduces an improved metaheuristics-based clustering with multihop routing protocol for underwater wireless sensor networks, named the IMCMR-UWSN technique. The major aim of the IMCMR-UWSN technique is to choose cluster heads (CHs) and optimal routes to a destination. The IMCMR-UWSN technique incorporates two major processes, namely the chaotic krill head algorithm (CKHA)-based clustering and self-adaptive glow worm swarm optimization algorithm (SA-GSO)-based multihop routing. The CKHA technique selects CHs and organizes clusters based on different parameters such as residual energy, intra-cluster distance, and inter-cluster distance. Similarly, the SA-GSO algorithm derives a fitness function involving four parameters, namely residual energy, delay, distance, and trust. Utilization of the IMCMR-UWSN technique helps to significantly boost the energy efficiency and lifetime of the UWSN. To ensure the improved performance of the IMCMR-UWSN technique, a series of simulations were carried out, and the comparative results reported the supremacy of the IMCMR-UWSN technique in terms of different measures.

## 1. Introduction

A large number of studies have been conducted on terrestrial sensor networks concerning several aspects, and currently, the underwater wireless sensor networks (UWSNs) have attracted growing interest from researchers [1,2]. They are different from the widely employed land-based sensor networks in terms of the cost of expensive sensors, the transmission system of acoustic signals, the dense deployment of sensors, the larger storage space to save maximal information, and the maximal power for transmission [3,4]. An UWSN consists of movable and stationary nodes that interact via acoustic networks. UWSN is utilized in various applications which have underwater environments, such as pollution monitoring, particularly the monitoring of the population of underwater flora and fauna, chemical waste, disaster prevention, examining the health of rare marine creatures, assisted navigation, mine reconnaissance, oil leakage detection, nutrient production, oceanographic data collection [5,6], distributed tactical surveillance, underwater military applications, target tracking, and detection. The researcher faces a lot of problems while working in UWSNs, such as longer propagation delays, narrow bandwidth, temporary loss of connectivity [7,8], harsh geographical atmosphere, shadow zones, constrained energy, attenuation, high bit error rates, and a comparatively smaller network scale.

Communication distance, energy consumption, and network life cycle are the three major considerations for designing UWSNs [9]. Topology control using the clustering model is the major solution to the problem of the UWSNs since it could balance the energy utilization, prolong the lifetime of the networks, and reduce communication interference [10]. The objective of clustering is to split the whole UWSN into different areas. In all the regions, sensors only interact with the cluster head (CH) within their own cluster. Earlier routing protocols were developed for certain layers, for example, design protocols for the transport or network layers [11]. This protocol entailed a layered protocol framework [12]. They do not take advantage of energy levels, joint optimization, and other causes, so total performance becomes insufficient [13,14,15]. Hence, the present study focuses on designing a protocol that could make use of data received by distinct layers, named the cross-layered routing protocol [16].

There is another issue that makes underwater transmission difficult. One of the problems is limited bandwidth. It has a direct effect on the communication rate of the network. Different sources of noise and water current lead to limited bandwidth in UWSN [17]. For longer distance transmission in UWSN, oceanic waves are utilized that could travel several kilometers at higher power and lower frequency [18]. The underwater network faces the loss of connectivity and a high bit error rate because of multi-path interference from oceanic networks. Because of the water temperature, multipath noise effect, and Doppler spread, underwater transmission is not reliable [19,20]. Furthermore, the multi-path effect causes the incoming signal fade. As a result, routing becomes costly and challenging in UWSN [21].

This study introduces an improved metaheuristics-based clustering with a multihop routing protocol for UWSN, named the IMCMR-UWSN technique. The IMCMR-UWSN technique intends to properly arrange the nodes into clusters and determine the shortest routes for inter-cluster data transmission in the UWSN [22,23]. To accomplish this, the IMCMR-UWSN technique designs the chaotic krill head algorithm (CKHA)-based clustering and self-adaptive glowworm swarm optimization algorithm (SA-GSO)-based multihop routing protocols [24,25,26]. Since multiple input parameters are used for the selection of CHs and optimum routes in UWSN, the overall network efficiency can be considerably improved. The experimental results of the IMCMR-UWSN technique are validated and the results are distinct aspects are inspected [27,28,29]. 

The rest of the paper is organized as follows. Section 2 provides a brief review of existing cluster-based routing techniques in UWSN. Next, Section 3 offers a detailed discussion of the proposed model. Then, Section 4 evaluates the performance of the proposed model, and Section 5 concludes the study.

## 2. Literature Review

Yadav and Kumar [30] proposed an optimum clustering for UWSN that is compliant with free space optical (FSO), acoustic, and electromagnetic (EM) wave-based transmission systems. In particular, the applicability of the above-mentioned methods for underwater transmission is examined and their efficiency is compared based on optimal clustering and energy utilization [31,32].

Fei et al. [33] proposed a hybrid clustering model based on moth–flame optimization (MFO) and fuzzy c-mean (FCM) techniques for improving the efficiency of the system. The concept is to create an energy-effective cluster by utilizing FCM and later using an optimization method to select an optimum CH within all the clusters [34,35].

In Song et al. [36], a dynamic hierarchical clustering data collection method based on multi-criteria decision making in 3D UWSNs was developed. First, the whole monitoring network is separated into different layers. In order to select a CH in all the layers, the multi-criteria decision-making of hierarchical fuzzy integration and the analytic hierarchy process (AHP) is adapted. Moreover, they utilized a sorting approach for the formation of a clustering topology model to resolve the problems. Next, they proposed an energy-balanced routing method between clusters [37,38].

Li et al. [39] proposed a clustering method based on the discrete particle swarm optimization (PSO) algorithm. Next, the clustering method they used was shown to keep UASN clustered, but with the CH moving around [40,41].

Yu et al. [42] designed an energy optimization clustering algorithm (EOCA) for the multihop underwater cooperative sensor networks (UWA-CSNs) because of the constrained energy source of the underwater sensors. The presented systems consider various factors, like the distance between the underwater sensors and sink nodes, the number of neighboring nodes, the RE of all the nodes, and the movement of the sensors produced by the ocean current [43,44].

Wang et al. [45] developed an energy utilization balanced protocol, called dynamic clustering k-means (DC-K-means)-based simplified balanced energy adaptive routing (S-BEAR), to extend the lifespan of underwater sensor networks (UWA-SN) by avoiding the energy hole and balancing the energy utilization of underwater sensors.

Omeke et al. [46] presented a novel approach called the distance- and energy-constrained k-means clustering system (DEKCS) for the selection of CH. The potential CH is chosen according to its residual battery level and location in the cluster. Then, the remaining energy threshold set for CHs is dynamically upgraded to guarantee that the network completely runs out of energy before it is disconnected.

Xiao et al. [47] proposed a genetic algorithm (GA)-based enhanced mutation operation, an encoding system, and a crossover operation. In addition, the back propagation neural networks (BPNN) used for data fusion are enhanced by adopting a momentum technique that could minimize energy utilization by decreasing the amount of transmitted data and eliminating the redundancy of data [48,49,50,51]. The existing studies and their descriptions are discussed in Table 1.

## 3. The Proposed Model

**Figure 1:**
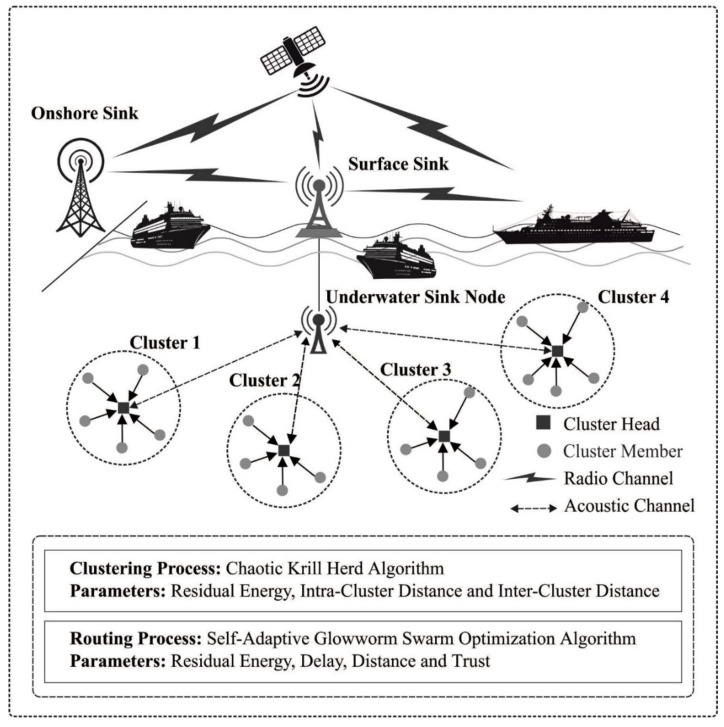
Overall process of the IMCMR-UWSN technique.

The purpose of this work is to develop a novel IMCMR-UWSN technique for maximizing the energy efficiency and longevity of UWSN. The IMCMR-UWSN technique led to the invention of the CKHA technique and formed the clusters effectively. Additionally, a new routing technique dubbed SA-GSO has been introduced and derived as a function for efficiently selecting base station routes (BS). The following sections discuss the operation of two significant sub-processes. The IMCMR-UWSN technique’s entire operation is depicted in Figure 1.

### 3.1. Energy Model

The underwater energy utilization method shown in [52] is used in the study. This study considered $P_{0}$ as the minimum power that a node needed to receive the packet, and minimum transmission power must attain $P_{0}A\left(l\right)$, whereas $A\left(l\right)$ represents an attenuation function. The energy utilization for receiving and transmitting is estimated as follows:

(1) $$E_{t}\left(l\right)\;=T_{t}P_{0}A\left(l\right)$$

(2) $$E_{r}=T_{r}P_{0}$$

(3) $$A\left(l\right)\;=l^{1.5}a^{l}$$

(4) $$a=10^{\alpha \left(f_{C}\right)/10}$$

(5) $$\alpha \left(f_{c}\right)\;=0.11\frac{f_{c}^{2}}{1+f_{c^{2}}}+44\frac{f_{c}^{2}}{4100+f_{c^{2}}}+2.75\times 10^{-4}f_{c}^{2}+0.003$$

in which $E_{t}\left(l\right)$ indicates the energy utilization to transmit, and $E_{r}$ indicates the energy utilized for receiving. $T_{t}$ represents the time for the node to transmit the packet, and $T_{r}$ shows the time to receive the packets. l denotes the distance among transmitting and receiving the nodes. $\alpha \left(f_{c}\right)$ represents the absorption coefficient in dB/km and $f_{c}$ shows the frequency in kHz.

### 3.2. Process Involved in CKHA-Based Clustering Technique

**Figure 2:**
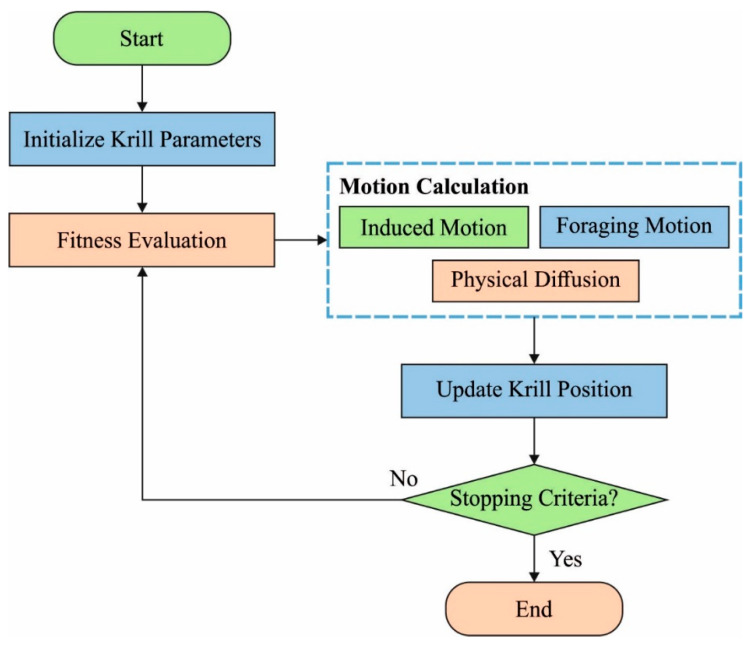
Flowchart of KH.

The sensor nodes are initially deployed in the target area and the startup process is initiated. Krill head (KH) is a generic stochastic optimization technique for solving global optimization problems. It is induced by the krill swarm’s activity [53]. While hunting for food and interacting with one another, the KH approach repeats three motions and follows the search direction, which enhances the objective function values. The flowchart of the KH approach is depicted in Figure 2. Three motions primarily characterize the time-based location:

- Foraging behaviour.
- Motion impacted by other krill.
- Physical diffusion.

The standard KH method adapts the Lagrangian algorithm as follows:

(6) $$\frac{dxi}{d_{t}}=N_{i}+F_{i}+D_{i’}$$

whereas $N_{i},$ $F_{i}$ and $D_{i}$ indicates the foraging motion, i.e., impacted by other krill and the physical diffusion of krill i, correspondingly. The primary motion $F_{i}$ consists of data about the preceding position and the existing food position:

(7) $$F_{i}=V_{f}\beta_{j}+w_{f}F_{i}^{old}$$

in which:

(8) $$\beta_{i}=\beta_{i}^{food}+\beta_{i}^{best}$$

the second move is dictated by the position of the food as well as the previous experience with the item in question and $V_{f}$ denotes the foraging speed, $w_{f}$ indicates the inertia weight of the movement within $\left(0,1\right)$ and is the final foraging movement.

The direction directed by the second motion $N_{i},$ $a_{i}$ is evaluated by: repulsive, target, and local effects. For a krill i, it is expressed by:

(9) $$N_{i}^{new}=N^{\;\max\;}a_{i}+w_{n}N_{i}^{old}$$

For the first motion, αi represents the direction of its motion and is determined by a target, a local influence, and a repulsive effect. Here $N^{\;\max\;}$ represents the maximal induced speed, $w_{n}$ dents the inertia weights of the second movement in $\left(0,1\right)$ and is the final movement impacted by other krill [54]. 

K-means clustering is a vector quantization method derived from signal processing that tries to split *n* observations into k clusters, with each observation belonging to the cluster with the nearest mean (cluster centers or cluster centroid), which serves as the cluster’s prototype.

To i-th krill, in fact, the physical diffusion is an arbitrary method. This movement includes oriented vector and maximal diffusion speed. The physical diffusion is expressed by:

(10) $$D_{i}=D^{\;\max\;}\delta ,\;$$

In the Equation, $D^{\;\max\;}$ denotes the maximal diffusion speed and δ indicates the oriented vector whose values are an arbitrary value among [−1, 1].

As per above, the time-based location from time t to t+Δt is expressed as follows:

(11) $$X_{i}\left(t+\Delta t\right)=X_{i}\left(t\right)+\Delta t\frac{dx_{i}}{dt}.$$

The chaotic concept is integrated with the krill head algorithm (KHA) to improve search efficiency and ensure convergence to the optimal solution [55]. Given that Chebyshev maps are the most often used chaotic behavioral maps, it is likely that chaotic sequences can be generated efficiently and quickly. Additionally, longer sequences are not required. Chebyshev maps utilized during the CKHA model changes the value of randomized parameter $\omega^{chebychev}=\left(\omega_{d},\;\omega_{f}\right)$ in KH.

Chebyshev map updates the parameter $\omega_{d}$ and $\omega_{f}$ according to the following equations:

(12) $$\omega_{j}^{chebychev}=\;\cos\;\left(j*\cos^{-1}\left(\omega_{j-1}^{chebychev}\right)\right)$$

The above equation generates a chaotic sequence in the zero and one range. For each independent performance $\omega_{0}^{chebychev}$ is randomly taken. The chaotic values $\omega_{j}^{chebychev}$ generated by a logistical map with three hundred runs and $\omega_{0}^{chebychev}=0.001$ is represented by. In the case of CKHA [22],

(13) $$N_{k}^{next}=N^{\;\max\;}\alpha_{k}+\omega_{d,j}^{chebychev}N_{k}^{present}$$

(14) $$F_{k}^{nexi}=V_{f}\beta_{k}+\omega_{f,j}^{chebychev}F_{k}^{previous}\;\;\;\;\;\;$$

According to UWSN’s energy utilization approach, the network’s energy consumption is influenced by the communication distance. In fact, the selection of CHs is an optimized problem, and the optimized problem’s motivation is to reduce the generating function’s main function. As a result, the distance between a sensor node and the CH of all clusters is expressed as follows:

(15) $$dist_{CM}=\overset{N_{j}}{\underset{i=1}{\sum }}d\left(CM_{ij},\;CH_{j}\right)\;\left(j=1,2,\;\ldots \;,\;c\right)\;\;\;\;$$

where $c_{M_{ij}}$ implies the member node from cluster j, $c_{H_{j}}$ refers to the CH from cluster j, and in all the clusters, there are Nj nodes.

The distance amongst a CH and BS is as follows:

(16) $$dist_{CB}=d\left(CH_{j},\;BS\right)$$

where BS refers to the place of BS. The energy utilization of network to broadcast the data packet is written as:

(17) $$E_{To}=\overset{c}{\underset{i=1}{\sum }}(E_{CH}^{i}+\overset{N_{i}}{\underset{j=1}{\sum }}E_{MEM}^{ij})\;$$

It may generate the primary function based on the three impacts mentioned above. Additionally, if disparities exist between the three factors on the scale, the normalization function can be used to eliminate them. The sigmoid function is used to convert the three factors to zero and one. Generally, the sigmoid function was presented as:

(18) $$S\left(x\right)=\frac{1}{1+e^{-x}}\;$$

However, it generates the main purpose is:

(19) $$F_{obj}=W_{1}^{*}dist_{CM}+W_{2}^{*}dist_{CB}+W_{3}^{*}E_{To}$$

where *W*_1_, *W*_2_, and *W*_3_ imply the weight co-efficient that is changed for determining the priority of 3 factors. Concurrently, W1+W2+W3=1

### 3.3. Process Involved in SA-GSO-Based Multihop Routing

The SA-GSO technique can be used efficiently during the routing phase to determine the ideal paths to the destination. GSO [23] is an intelligently tuned technique that relies on the glow-worm light being used as a signal to attract another glow-worm. This strategy utilizes a randomly distributed group of glow-worms from the solution space. Each glow-worm is a viable solution denoted by their location. The glow-worm with the highest luminosity attracts the glow-worm with the lowest luminosity. This way, the technique’s global optimization is accomplished. The following are the fundamental phases:

Step 1. Initialization the fundamental parameter of GSO. This parameter contains the population size g, fluorescein volatilization factor ρ, fluorescein upgrade rate γ, upgrade rate β of the dynamic decision field, the group of glowworms $N_{i}\;\left(t\right)$ from the decision field, threshold $n_{t}$ for the number of glow-worms from the neighborhood, perception radius $r_{s}$, and move step s.

Step 2. The fitness value of glow-worm i at $t^{th}$ iteration was changed as to the fluorescein value with the subsequent equation:

(20) $$l_{i}\left(t\right)=\left(1-\rho \right)l_{i}\left(t-1\right)+\gamma J\left(X\left(t\right)\right)$$

where ρ refers to the fluorescein decompose constants going from zero and one, and γ demonstrates the fluorescein improvement constant.

Step 3. All the glow-worms choose individuals with superior brightness than themselves in their dynamic decision radius $r_{d}^{i}\left(t\right)$ for the procedure of their neighbor set $N_{i}\left(t\right)$.

Step 4. Compute the probability $p_{ij}\left(t\right)$ of glow-worm $X_{i}\left(t\right)$ affecting the glow-worm $X_{j}\left(t\right)$ from their dynamic decision radius by Equation (21):

(21) $$p_{ij}\left(t\right)=\frac{l_{j}\left(t\right)-l_{i}\left(t\right)}{\sum_{k\in N_{i}\left(t\right)}l_{k}\left(t\right)-l_{i}\left(t\right)}$$

Step 5. Upgrade the place of glow-worm $X\left(t\right)$ in Equation (22):

(22) $$X_{i}\left(t+1\right)=X_{i}\left(t\right)+s\times \left[\frac{X_{j}\left(t\right)-X_{i}\left(t\right)}{\left|\left|X_{j}\left(t\right)-X_{i}\left(t\right)\right|\right|}\right]$$

Step 6. Upgrade the dynamic decision radius of glow-worm $X\left(t\right)$ in Equation (23):

(23) $$r_{d}^{i}\left(t+1\right)=\;\min\;\left\{r_{s},\;\max\;\left\{0,\beta \times \left(n_{t}-\left|N_{i}\left(t\right)\right|\right)\right\}\right\}$$

Generally, in the GSO algorithm, predefined values are allotted to the step size as a fixed value. Since the proper choice of step size is important for effective outcome, in this study, two factors influencing the step size are considered, namely the number of rounds and distance between the glow-worm and optimal glow-worm at the $ni^{th}$ round. If the $i^{th}$ glow-worms are located farther from optimum solutions, the step size becomes high, otherwise, it becomes small. At the $ni^{th}$ round, when the $i^{th}$ glow-worm indicates the optimal one, its step size results in 0. After examining the impact of moving step size on the GSO algorithm [56,57], the SA-GSO algorithm (Algorithm 1) is derived by the use of self-adaptive step size formulation, as given below:

(24) $$s_{i}\left(t\right)=D_{i}\left(t\right)\cdot \left(len\left(e-\frac{t}{N_{t}}\right)\right)\left\|x_{i}\left(t\right)-x_{b}\left(t\right)\right\|$$

where each $x_{i}\left(t\right)$ is assigned to exactly one $s_{i}\left(t\right)$, even if it could be assigned to two or more of them, where $D_{i}\left(t\right)$ implies an arbitrary number in uniform distribution, $N_{t}$ denotes maximum iterations, and $x_{b}\left(t\right)$ indicates the location of the optimal glow-worm at the $t^{th}$ round.

**Algorithm 1:** Pseudocode of GSO Algorithm.Initialization: *m* dimension Initialization: *n* glowworms Let *s* be step size Let
$x_{i}(t)$ indicates the location of glow-worm *t* at time instant *t*
Deploy agents in an arbitrary way
$deploy-agents_{–}randomly;$
for $i=1\;\mathrm{to}\;n\;\mathrm{do}\;\ell_{i}(0)=\ell_{0}$

$r_{d}^{i}\left(0\right)=r_{0}$
Consider highest number of iterations = max_iter;
assume t=1;
while $\left(t\leq \max\_iter\right)$ do:
  { for every glowworm *i* do
$\ell_{i}\left(t\right)=\left(1-\rho \right)l_{i}\left(t-1\right)+\gamma \;Fitness\;\left(x_{i}\left(t\right)\right);$
for every glow-worm *i* do {
$N_{i}\left(t\right)=\{j:d_{ij}\left(t\right)<r_{d}^{i}\left(t\right);\ell_{i}\left(t\right)<\ell_{j}\left(t\right)\};$
for every glow-worm $j\in N_{i}\left(t\right)$ do:
$p_{ij}\left(t\right)=\frac{\ell_{j}\left(t\right)-\ell_{i}\left(t\right)}{\Sigma p\in N_{i}\left(t\right)\ell_{p}\left(t\right)-\ell_{i}\left(t\right)};$

$j=select\_glowworm\left(\vec{p}\right);$

$x_{i}\left(t+1\right)=x_{i}\left(t\right)+step\left(\frac{x_{j}\left(t\right)-x_{i}\left(t\right)}{\left\|x_{j}\left(t\right)-x_{i}\left(t\right)\right\|}\right)$

$r_{d}^{i}\left(t+1\right)=\min\;\left\{r_{sy},\;\mathrm{maximumm}\;\left\{0,\;r_{d}^{i}\left(t\right)+\beta \left(n_{t}-\left|N_{i}\left(t\right)\right|\right)\right\}\right\};$
 }
t←t+1;
  }

The SA-GSO algorithm is derived by considering parameters like trust, energy, delay, and distance in order to determine the optimal route for data transfer between sender and destination via CHs in UWSN as discussed in Algorithm 1. Because the fitness with the highest value is considered the optimal path, the maximal fitness can be computed using the largest trust value, the shortest latency, the highest energy, and the shortest distance. The maximal fitness function can be evaluated using the following formulas:

(25) $$B=\frac{1}{4}\left[b+\left(1-h\right)+\left(1-\beta \right)+\mu \right]$$

(26) $$b=\frac{1}{a}\overset{a}{\underset{K-1}{\sum }}b_{K}$$

(27) $$h=\frac{a}{m}\;$$

(28) $$\beta =\frac{1}{a^{2}\times \eta }\overset{a}{\underset{K=1}{\sum }}\frac{\sum_{T=1\;}^{a}\;\beta \left(K,\;T\right)}{T=K+1}$$

(29) $$\mu =\frac{1}{3a^{2}\times \eta }\overset{a}{\underset{K=1}{\sum }}\left[DT+RT+HT\right]\;$$

whereas B represents fitness function, b indicates energy, h denotes delay, β represents the distance, μ shows trust, and η indicates the normalization factor.

## 4. Experimental Validation

**Figure 3:**
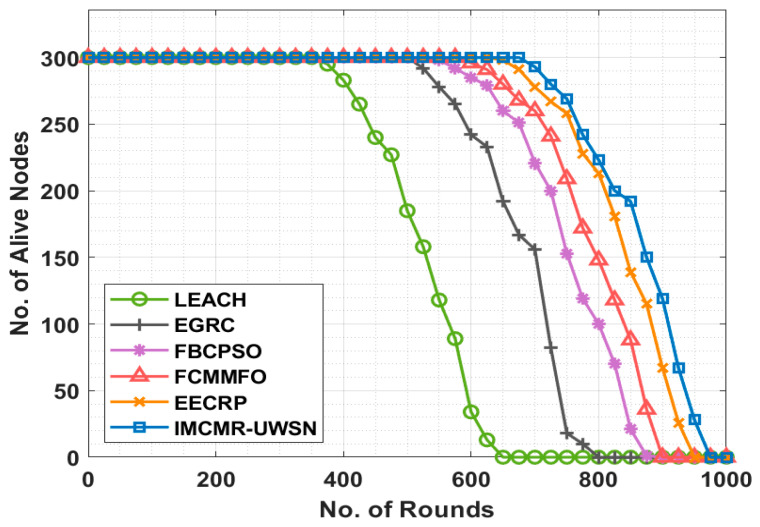
NAN analysis of the IMCMR-UWSN technique.

This section examines the performance of the IMCMR-UWSN technique using contemporary approaches in a variety of contexts. Figure 3 compares the IMCMR-UWSN technique’s number of alive nodes (NAN) analysis to previous techniques over several iterations. The experiment was conducted with the 2018a edition of MATLAB testing, which was performed on a 5th generation, core i5 system with 8 GB RAM. This experimentation was carried out with different grid sizes, which ranged from 500 m to 2000 m. This is also true for the tests. The number of nodes used ranged from 0 to 300, and the range of the nodes’ transmission range changed from 25 m to 200 m. Nodes are supposed to stay in the same place or move very slowly because of water flow. The experimental results indicated that the low-energy adaptive clustering hierarchy (LEACH) methodology produced suboptimal outcomes with the smallest possible NAN. Following that, the EGRC approach achieved a somewhat higher NAN value than the LEACH protocol. Accordingly, the forward backward conventional particle swarm optimization (FBCPSO) and FCMMFO approaches resulted in a somewhat closer NAN after numerous cycles. While the EECRP methodology attempted to achieve a reasonable NAN in comparison to the other ways, the disclosed IMCMR-UWSN strategy outperformed them all in terms of NAN.

**Figure 4:**
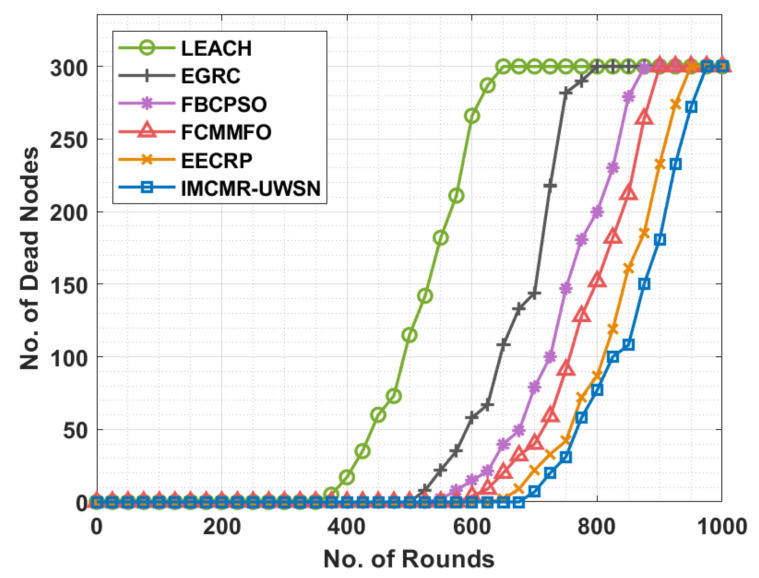
NDN analysis of the IMCMR-UWSN technique.

Figure 4 compares the IMCMR-UWSN technique with other strategies in terms of the number of dead nodes (NDN). The graphic demonstrated the LEACH protocol’s ineffectiveness in comparison to other approaches with a greater NDN. Additionally, the EGRC approach achieved a somewhat lower NDN concentration than the LEACH protocol. Accordingly, over numerous iterations, the FBCPSO and FCMMFO approaches resulted in a moderately closer NDN. While the EECRP technique achieved a slight advantage in terms of NDN over the other ways, the provided IMCMR-UWSN technique outperformed the previous techniques with the least amount of NDN. Calculate the distance d (ni, CHp, k) between node n_i_ and all CHs, CHp, k for all n_i_ = 1, 2, …, N.

**Figure 5:**
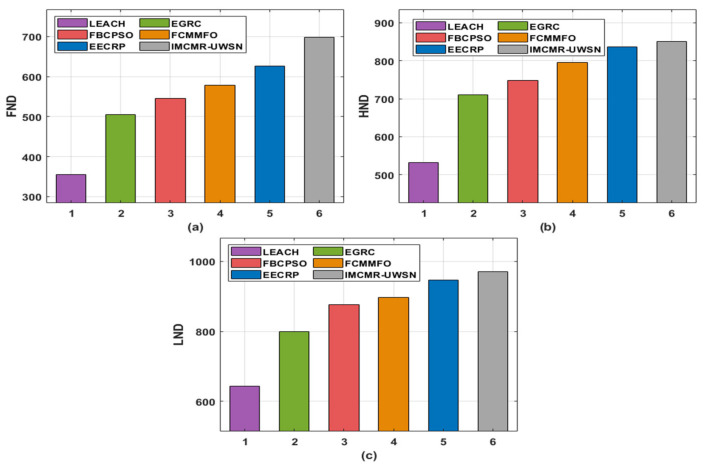
NLT analysis of IMCMR-UWSN technique with existing methods (**a**) FND; (**b**) HND; (**c**) LND.

Table 2 and Figure 5 illustrate the IMCMR-UWSN technique’s named data networking (NDN) analysis using modern techniques. The testing results validated the IMCMR-UWSN technique’s increased NLT by extending the rounds of first node death (FND), half node death (HND), and last node death (LND) (LND). In terms of FND, the IMCMR-UWSN approach achieved FND after 698 rounds, whereas the LEACH, EGRC, FBCPSO, FCMMFO, and EECRP strategies achieved FND after 355, 505, 546, 578, and 626 rounds, respectively.

Furthermore, when HND is considered, the IMCMR-UWSN technique achieves HND at an increased round count of 875 but the LEACH, EGRC, FBCPSO, FCMMFO, and EECRP procedures achieve HND at reduced round counts of 532, 711, 748, 796, and 837, respectively. Furthermore, when LND is considered, the IMCMR-UWSN technique achieved LND at 698 rounds but the LEACH, EGRC, FBCPSO, FCMMFO, and EECRP techniques reached LND at 643, 799, 876, 896, and 846 rounds, respectively.

**Figure 6:**
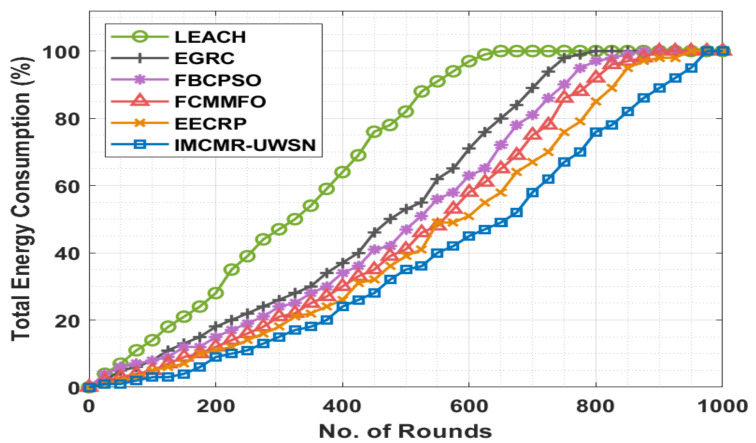
TEC analysis of the IMCMR-UWSN technique.

Figure 6 compares the IMCMR-UWSN approach to various techniques in detail in terms of total energy consumption (TEC). The graphic demonstrated the LEACH protocol’s ineffectiveness against other approaches with a maximum TEC. Additionally, the EGRC method achieved a somewhat lower TEC than the LEACH technique. Additionally, the FBCPSO and FCMMFO techniques resulted in a slightly closer TEC across a large number of iterations. However, while the EECRP approach achieved a somewhat higher TEC than the other approaches, the reported IMCMR-UWSN methodology outperformed current techniques with a lower TEC.

**Figure 7:**
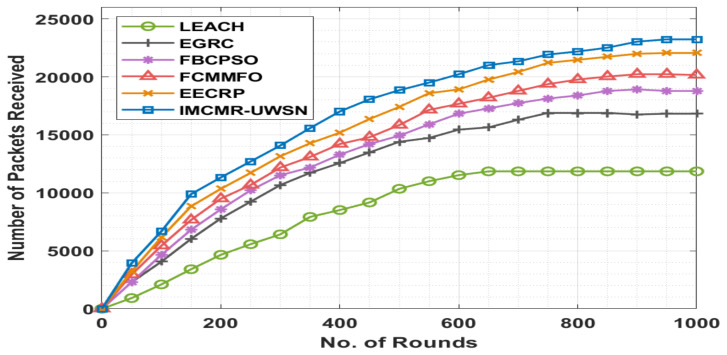
NOPR analysis of the IMCMR-UWSN technique.

Table 3 and Figure 7 compare the IMCMR-UWSN method’s number of packets received (NOPR) analysis to other methods in various iterations. The experimental results demonstrated that the LEACH approach achieved worse results with a lower NOPR. Following that, the EGRC technique achieved a slightly higher NOPR than the LEACH protocol. Following that, multiple rounds of the FBCPSO and FCMMFO techniques resulted in a moderately closer NOPR. While the EECRP strategy attempted to achieve a fair NOPR in comparison to the other approaches, the disclosed IMCMR-UWSN methodology outperformed them all with a greater NOPR. 

By examining the tables and figures above, it is clear that the IMCMR-UWSN technique has been demonstrated to be an excellent instrument for achieving maximum energy efficiency and lifetime in the UWSN environment. The preceding findings compare the IMCMR-UWSN method’s number of packets received (NOPR) analysis against existing methods in various iterations. The experimental results demonstrated that the LEACH technique performed poorly with a lower NOPR in all rounds. Following that, the EGRC model achieved a somewhat higher NOPR than the LEACH methodology. Following that, across numerous rounds, the FBCPSO and FCMMFO approaches resulted in a moderately closer NOPR. With a larger NOPR, the proposed IMCMR-UWSN model achieved the highest performance.

## 5. Conclusions

The purpose of this work is to develop a novel IMCMR-UWSN technique for maximizing the energy efficiency and longevity of UWSN. The IMCMR-UWSN technique led to the invention the CKHA technique and formed the clusters effectively. Additionally, a new routing technique termed the SA-GSO technique was introduced and identified as a function for selecting the optimal routes to BS. The use of numerous CH input parameters and effective route selection contributes to the network’s overall performance improvement. The experimental results analysis for the IMCMR-UWSN technique is validated, and the findings are examined under a variety of different circumstances. The complete comparison analysis demonstrated the IMCMR-UWSN technique’s superior performance to other contemporary techniques. In the future, the IMCMR-UWSN technique’s energy efficiency can be increased further through the design of data aggregation methodologies. Additionally, resource allocation strategies based on metaheuristic algorithms can be created to efficiently allocate resources.

## Figures and Tables

**Table 1:** The state-of-the-art clustering with multihop routing protocol for underwater wireless sensor networks.

Ref. No.	Methodology	Description	Pros	Cons
[30]	FSO communication compliant	Free space optical (FSO), acoustic, and electromagnetic (EM) waves-based transmission systems.	No extra packet transmission occurs due to the use of the priority value.	High error ratepacket redundancy.
[33]	Fuzzy c-means and moth–flame optimization.	Creates energy-effective cluster by utilizing FCM and later uses optimization method for selecting an optimum CH within all the clusters.	Reduces energy consumption. Enhances network lifetime.	Time delay packet loss.
[36]	Multiple criteria decision-making.	Sorting approach is utilized for the formation of clustering topology model to resolve the problems.	Reduces network delay. High packet delivery.	Low throughput. Less reliability.
[13]	Improved particle swarm optimization algorithm.	Discrete particle swarm optimization (PSO) algorithm applied for effective clustering model.	Throughput is high. Reliability in clustering process. Efficient routing mechanism.	Network life time packet delivery ratio.
[39]	Underwater acoustic cooperative sensor networks.	Improves the constrained energy source of the underwater sensors.	Low energy consumptions. Improving lifetime of network.	Time delay and packet loss.
[42]	Multi-hop transmission for underwater acoustic sensor networks.	Extends lifespan of underwater sensor networks by avoiding the energy hole and balancing the energy utilization of underwater sensors.	Packet delivery ratio is high. Reliable transmission.	Not suitable for deep water area networks. Dead nodes formation.
[45]	Dynamic clustering protocol.	Dynamically upgrades the energy threshold set for CHs to guarantee that the network communication.	Avoiding of Packet collision Reducing network delay. Optimal path routing.	Sensor communication failure.
[47]	Data fusion and genetic algorithms.	Used for data fusion is enhanced by adopting a momentum technique that could minimize energy utilization.	High throughput. Reliable transmission.	Maximization of connection time and network delay.

**Table 2:** Network lifetime analysis of the IMCMR-UWSN technique.

Methods	FND	HND	LND
LEACH	355	532	643
EGRC	505	711	799
FBCPSO	546	748	876
FCMMFO	578	796	896
EECRP	626	837	946
IMCMR-UWSN	698	875	970

**Table 3:** Number of packets received analysis of the IMCMR-UWSN technique with different rounds.

No. of Rounds	LEACH	EGRC	FBCPSO	FCMMFO	EECRP	IMCMR-UWSN
0	0	0	0	0	0	0
50	922	2296	2296	2951	3212	3932
100	2100	4063	4652	5437	6157	6680
150	3409	6026	6811	7662	8839	9886
200	4652	7792	8578	9494	10,344	11,326
250	5568	9232	10,213	10,671	11,718	12,700
300	6418	10,671	11,522	12,176	13,158	14,074
350	7923	11,718	12,176	13,092	14,270	15,579
400	8512	12,569	13,289	14,205	15,186	17,018
450	9167	13,485	14,205	14,793	16,364	18,065
500	10,344	14,401	14,924	15,840	17,411	18,850
550	10,999	14,728	15,906	17,149	18,588	19,504
600	11,522	15,448	16,822	17,672	18,915	20,224
650	11,849	15,644	17,280	18,196	19,766	21,009
700	11,849	16,298	17,738	18,785	20,420	21,336
750	11,849	16,887	18,130	19,374	21,206	21,925
800	11,849	16,887	18,392	19,766	21,467	22,187
850	11,849	16,887	18,785	20,028	21,729	22,514
900	11,849	16,756	18,915	20,224	21,991	23,038
950	11,849	16,822	18,785	20,224	22,056	23,234
1000	11,849	16,822	18,785	20,159	22,056	23,234

## Data Availability

The study did not report any data.
